# Supplementary material for: From sequence to enzyme mechanism using multi-label machine learning
Source: BMC Bioinformatics. 2014 May 19;15:150. doi: 10.1186/1471-2105-15-150 (PMC4229970; doi:10.1186/1471-2105-15-150)
Supplement: Additional file 2 — Java code of ml2db. Additional file ml2db_code.tar.gz contains the Java source code to run the multi-label machine learning experiments and save the results to database. The code’s Javadoc is included. [file 1471-2105-15-150-S2.zip › additional file 2/ml2db/ml2db/doc/index-files/index-5.html]

E-Index


JavaScript is disabled on your browser.


- Overview
- Package
- Class
- Use
- Tree
- Deprecated
- Index
- Help

- Prev Letter
- Next Letter

- Frames
- No Frames

- All Classes

A B C D E F G H I J K L M N O P Q R S T U V W X 


## E

EMPTY\_INSTANCE\_ATTRIBUTES\_STRING - Static variable in class main.ml2db.mulan.mulanarff.IdMultiLabelInstances


EMPTY\_STRING - Static variable in class main.ml2db.mulan.mulanarff.IdMultiLabelInstances


end\_graph() - Method in class main.ml2db.graphviz.GraphViz
:   Returns a string that is used to end a graph.

END\_TIMESTAMP - Static variable in class main.ml2db.al.database.AlExperimentsTable
:   time at end of computation

END\_TIMESTAMP - Static variable in class main.ml2db.mulan.learn.crosseval.EvaluationResultsTable
:   time at end of computation

equals(IdInstance) - Method in class main.ml2db.mulan.mulanarff.IdInstance


equals(Instance) - Method in class main.ml2db.mulan.mulanarff.IdInstance


EUCLIDEAN\_DISTANCE\_NAME - Static variable in class main.ml2db.al.ActiveLearning


evaluate(int, IdMultiLabelInstances, MultiLabelLearner, boolean, boolean, boolean) - Method in class main.ml2db.mulan.learn.crosseval.CrossEvaluator
:   Evaluates (runs a cross validation)

evaluate(MultiLabelLearner, MultiLabelInstances, boolean, PredictionsSaver) - Method in class main.ml2db.mulan.learn.evaluation.EvaluatorNoAUC
:   Evaluates a `MultiLabelLearner` on given test data set.

evaluate(MultiLabelLearner, MultiLabelInstances, List<Measure>, boolean, PredictionsSaver) - Method in class main.ml2db.mulan.learn.evaluation.EvaluatorNoAUC
:   Evaluates a `MultiLabelLearner` on given test data set using
    specified evaluation measures

evaluateAndSave() - Method in class main.ml2db.al.learning.AlExperimenter


evaluateAndSave(int, boolean, boolean, boolean) - Method in class main.ml2db.mulan.learn.crosseval.CrossExperimenter
:   Cross validates returns evaluation results.

evaluateAndSave(boolean, boolean) - Method in class main.ml2db.mulan.learn.traintest.TrainTestExperimenter


EVALUATION\_TABLE\_NAME - Static variable in class main.ml2db.al.database.AlEvaluationTable


EvaluationMetricsTest - Class in test.ml2db.mulan.learn.traintest
:   Tests the evaluation metrics results: accuracy, precision, recall etc.

EvaluationMetricsTest() - Constructor for class test.ml2db.mulan.learn.traintest.EvaluationMetricsTest


EvaluationParameters - Class in main.ml2db.mulan.learn.evaluation
:   Contains the evaluation parameters (micro-macro precision, micro-macro
    accuracy etc.) of a Multi label Mulan experiment.

EvaluationParameters() - Constructor for class main.ml2db.mulan.learn.evaluation.EvaluationParameters


EvaluationParametersTest - Class in test.ml2db.mulan.learn
:   Class

EvaluationParametersTest() - Constructor for class test.ml2db.mulan.learn.EvaluationParametersTest


EvaluationResultsTable - Class in main.ml2db.mulan.learn.crosseval
:   Gives the names and table field names for multi-label specific evaluation
    measures (such as micro-accuracy, macro-accuracy, hamming loss etc.)

EvaluationResultsTable(DbManager) - Constructor for class main.ml2db.mulan.learn.crosseval.EvaluationResultsTable


EvaluationResultsTableTest - Class in test.ml2db.mulan.learn
:   Class

EvaluationResultsTableTest() - Constructor for class test.ml2db.mulan.learn.EvaluationResultsTableTest


EvaluatorNoAUC - Class in main.ml2db.mulan.learn.evaluation
:   Prepares the evaluation measures taking into consideration the dataset size.

EvaluatorNoAUC(CrossExperimenter, boolean) - Constructor for class main.ml2db.mulan.learn.evaluation.EvaluatorNoAUC


EXPECTED\_NUMBER\_OF\_UNIQUE\_ATTRIBUTES - Static variable in class main.ml2db.ProjectParameters
:   used to optimise the pool of attributes strings

EXPECTED\_NUMBER\_OF\_UNIQUE\_INSTANCES - Static variable in class main.ml2db.ProjectParameters
:   used to optimise the pool of instances strings

EXPERIMENT\_ID - Static variable in class main.ml2db.al.database.AlDbCreator
:   the table column to store the unique experiment identifier

EXPERIMENT\_ID - Static variable in class main.ml2db.mulan.predict.PredictionsRecordTable
:   the identifier of the prediction experiment (algorithm, data, start time
    etc.) this record belongs to

ExperimentDetails - Class in main.ml2db.al.learning
:   Manages the data for an active learning experiment: experiment id, arff file
    used, algorithm for training/testing, type of distance used (Euclidean etc.),
    type of utility metric used to pick the next query instance to be labelled,
    timestamp etc.

ExperimentDetails(IdMultiLabelInstances, MulanLearner, String, DbManagers, UtilityMetric, String) - Constructor for class main.ml2db.al.learning.ExperimentDetails
:   With set of unlabelled instances (no arff record identifier)

ExperimentDetails(int, int, MulanLearner, String, DbManagers, UtilityMetric, String) - Constructor for class main.ml2db.al.learning.ExperimentDetails
:   Both labelled and unlabelled arff record identifier.

ExperimentDetails(int, MulanLearner, String, DbManagers, UtilityMetric, String) - Constructor for class main.ml2db.al.learning.ExperimentDetails
:   Only Unlabelled arff record identifier.

ExperimentDetailsTest - Class in test.ml2db.al
:   Class

ExperimentDetailsTest() - Constructor for class test.ml2db.al.ExperimentDetailsTest


Experimenter - Class in main.ml2db.mulan.learn
:   An object to run a crossevaluation or train-test evaluation experiment.

Experimenter() - Constructor for class main.ml2db.mulan.learn.Experimenter


ExperimenterTest - Class in test.ml2db.mulan.learn
:   Class

ExperimenterTest() - Constructor for class test.ml2db.mulan.learn.ExperimenterTest


experimentId() - Method in class main.ml2db.mulan.predict.PredictionsSaver


EXPERIMENTS\_TABLE\_NAME - Static variable in class main.ml2db.al.database.AlExperimentsTable
:   the name for the table to store the evaluation results

EXPERIMENTS\_TABLE\_NAME - Static variable in class main.ml2db.mulan.learn.crosseval.EvaluationResultsTable
:   the name for the table to store the evaluation results

experimentTable() - Method in class main.ml2db.mulan.predict.PredictionsSaver

A B C D E F G H I J K L M N O P Q R S T U V W X

- Overview
- Package
- Class
- Use
- Tree
- Deprecated
- Index
- Help

- Prev Letter
- Next Letter

- Frames
- No Frames

- All Classes
